# Role of Sexuality in the Quality of Life of Palliative Care Patients: A Narrative Review

**DOI:** 10.7759/cureus.112018

**Published:** 2026-07-03

**Authors:** Walter Iván Díaz Chamba, Diego Antonio Mena Noroña, Katherine Michelle Andrade Trávez, Andrea Verónica Díaz Chamba, Jazmín karina Farinango Balseca

**Affiliations:** 1 General Medicine, Pontificia Universidad Católica del Ecuador, Quito, ECU; 2 Faculty of Medical Sciences, Universidad Central del Ecuador, Quito, ECU; 3 Nursing, Hospital Marco Vinicio Iza, Quito, ECU; 4 Nutrition, Universidad Iberoamericana del Ecuador, Quito, ECU

**Keywords:** palliative care, patients needs, quality of life, sexual dysfunction, sexuality

## Abstract

This literature review identifies patients' experiences regarding sexuality in palliative care (PC) settings, the tools available for its assessment, and how healthcare professionals address these needs. A literature review was conducted through a comprehensive search of articles published during the last decade in the PubMed, ScienceDirect, and Google Scholar databases using predefined keywords. Seventeen studies were selected based on strict criteria of relevance, clinical significance, and psychosocial applicability to establish the argumentative foundation of this review. The findings revealed that the primary needs related to sexuality centered on the possibility of establishing effective communication with healthcare professionals, as sexuality remains an important aspect of quality of life for most patients receiving PC, regardless of disease stage. However, this dimension is rarely addressed in clinical practice. Furthermore, healthcare professionals often report feeling insufficiently trained and inadequately prepared to discuss these issues with patients, despite the availability of assessment tools such as the PLISSIT (Permission, Limited Information, Specific Suggestions, and Intensive Therapy) model. Sexual health constitutes an essential component of PC; however, its assessment and management remain limited due to sociocultural and healthcare-related barriers. The implementation of standardized protocols and continuous professional training strategies may facilitate its integration into clinical practice.

## Introduction and background

Cicely Saunders was a pioneer in palliative care (PC). She based her principles on treating the sick with humanity and compassion, so that they could live as fully and comfortably as possible [1]. Globally, the need for PCs is increasing rapidly. By 2060, there is projected to be an 87% global increase in conditions that cause severe suffering, such as cancer and dementia, and that are eligible for PC [2]. Furthermore, PC is essential for the world’s aging population. An estimated 20 million people require PC each year, 69% of whom are adults aged 60 years or older [3].

The early implementation of PC, whether at the onset of symptoms or at the time of diagnosis [4], has a positive impact on patients and healthcare services, as several studies have shown. For terminally ill patients with a life expectancy of one year or less, a randomized study evaluating home-based care showed a significant reduction in the use of healthcare services, decreasing emergency department visits by 13% (20% vs. 33%, *P* = 0.01) and hospitalizations by 23% (36% vs. 59%, *P* < 0.001) compared with medical care without PC [5]. In another oncology-specific study involving patients with lung cancer, the early integration of PC demonstrated a clear benefit in survival, prolonging survival by 2.7 months compared with oncological care without PC (11.6 vs. 8.9 months). This same study also associated early PC with a lower prevalence of depressive symptoms after a 12-week follow-up (16% vs. 38%, *P* = 0.01) [6]. Furthermore, a controlled trial in patients with advanced conditions such as congestive heart failure, chronic obstructive pulmonary disease, or cancer shows that consultations with a specialized PC team significantly reduce anxiety (*P* = 0.01) [7].

Within this framework of comprehensive care, sexuality is widely recognized as a fundamental dimension of the human experience throughout the lifespan. Its expression helps alleviate suffering and maintain interpersonal connections in the face of serious illness, according to the subjective experiences reported by patients themselves [8]. Although it is often mistakenly assumed that sexuality refers solely to sexual intercourse, this dimension goes beyond that and encompasses areas such as gender roles and orientations, eroticism, pleasure, and intimacy, which can be experienced and expressed through thoughts and feelings [9]; in this context, it is necessary to conceptualize the term intimacy as a multidimensional intra- and interpersonal process that involves physical, emotional, and spiritual connection [10]. It is essential to distinguish these concepts from sexual function, which refers to the physiological capacity to respond to stimulation; any impairment of this capacity leads to sexual dysfunction. While emotional support and the management of symptoms such as pain form the foundation of psychosocial well-being in PC, sexuality is often overlooked in care protocols; patients tend to assume that if the doctor does not ask about it, it is because it is not important. Sexuality is linked to identity, dignity, and well-being; for this reason, it must be addressed from a different perspective than other symptoms, so that specific interventions can be carried out. 

Despite its importance, the existing literature reveals a significant gap. Most previous reviews have focused on cancer populations, estimating that the prevalence of sexual dysfunction among cancer patients of both sexes ranges from 40% to 100%, particularly in breast cancer, gynecologic cancers, prostate cancer, testicular cancer, bladder cancer, and colorectal cancer; treatment may include chemotherapy, radiation therapy, immunotherapy, or surgery, depending on the type of cancer [11]. This has led to the neglect of the sexuality of people with other chronic degenerative diseases, creating a knowledge gap regarding PC for chronic non-oncological conditions, such as heart failure, kidney disease, or Parkinson’s disease, among others. For this reason, there is a need to bridge this knowledge gap to enable a comprehensive approach to patient care.

This gap in the literature reflects everyday clinical practice, as the scientific literature shows that sexuality remains a neglected aspect of medical consultation; for example, evidence indicates that the main reasons for not addressing sexuality include the patient not asking about it, limited consultation time, and the presence of a third party during the medical consultation [12].

Continuing education programs can improve clinical practice related to sexuality, as healthcare professionals often report that they do not feel sufficiently trained to address these issues during consultations [13] or simply believe that treating other symptoms takes priority over sexuality.

Addressing sexuality in palliative care requires validated tools to identify patients' specific needs. Among the intervention models, the PLISSIT framework (Permission, Limited Information, Specific Suggestions, and Intensive Therapy) stands out as a structured model designed to facilitate clinical communication and patient referral; it is described in detail in a later section [14]. For diagnostic assessment, several instruments have been developed, including the the Sexual Symptoms Checklist (SSCL), a brief screening tool for women following a cancer diagnosis [15]; the Female Sexual Function Index (FSFI), which assesses domains such as desire, arousal, and orgasm, along with its six-item abbreviated version [16]; and the Patient-Reported Outcomes Measurement Information System Sexual Function and Satisfaction Measure (PROMIS SexFS), which provides a comprehensive assessment of sexual function for both women and men . For the male population, available tools include the International Index of Erectile Function (IIEF) [17,18] and its abbreviated version, the International Index of Erectile Function-5 (IIEF-5), also known as the Sexual Health Inventory for Men (SHIM) [19], as well as the Expanded Prostate Cancer Index Composite (EPIC), which assesses sexual, urinary, bowel and hormonal domains [20]. Given the high prevalence of cancer, validated instruments are essential for assessing patient-reported outcomes. The European Organisation for Research and Treatment of Cancer Quality of Life Questionnaire-Sexual Health 22 (EORTC QLQ-SH22) was specifically developed to evaluate quality of life and sexual health in this population [21].

This review aims to highlight that addressing sexuality in PC practice is a fundamental, feasible, and modifiable pillar for promoting a person’s overall well-being. Through an analysis and synthesis of the existing literature, covering both oncology and non-oncology populations, this study evaluates the multidimensional impact of sexuality, addresses the main educational barriers faced by healthcare professionals, and describes intervention and education tools, such as the PLISSIT model, which is described in detail in a later section. Ultimately, this paper seeks to promote person-centered care, rather than disease-centered care, and break the clinical silence surrounding this intrinsic dimension of all human beings.

## Review

Methodology

The objective of this study is to conduct a narrative literature review to analyze the relevance of addressing sexuality as an integral component in improving the quality of life of patients receiving PC. To establish this framework, a rigorous, reproducible, and comprehensive bibliographic search was executed between May 15 and May 30, 2026. This comprehensive search targeted high-impact databases specializing in clinical and psychosocial health, PubMed, ScienceDirect, and Google Scholar, focusing primarily on scientific articles published during the last decade to ensure contemporary clinical relevance.

To address the relationship between sexual health and well-being in PC, specific Medical Subject Headings (MeSH) terms and title/abstract keywords were combined via Boolean operators using the following explicit search strings: ("palliative care" OR "terminal illness" OR "end-of-life care") AND ("sexuality" OR "sexual dysfunction" OR "intimacy") AND ("quality of life" OR "patient needs"). Language restrictions were configured to retrieve full-text manuscripts published exclusively in English, bounding the Google Scholar screening to the first 100 most relevant results per query combination. Studies were eligible based on predefined inclusion criteria (peer-reviewed primary quantitative, qualitative, or mixed-method research, and narrative/scoping reviews exploring the experiences or barriers of adult palliative patients, partners, or healthcare professionals), while single-case reports, clinical trial protocols, letters, abstract-only publications, or fertility/pediatric-focused studies were excluded. Following this exhaustive screening process, 17 key studies were included as the argumentative foundation of this work, which were meticulously analyzed according to their design: five quantitative studies (four cross-sectional surveys and one prospective longitudinal cohort evaluating patient-partner dyads), eight qualitative studies (five patient-centered and three professional-centered cohorts using interviews and thematic analyses), two mixed-methods studies evaluating rapid screening tools, and two literature/scoping reviews.

Although the primary literature search focused on the last 10 years, seminal historical studies published prior to 2016 were intentionally preserved and included based on explicit criteria of paradigm continuity and theoretical necessity. To prevent selection bias, a study was defined as *seminal* and selected only if it met at least one of the following objective thresholds: it established foundational international conceptual frameworks for PC or health-related quality of life; or it provided the primary validation or historical baseline for clinical assessment models and screening tools utilized within the review. These milestone publications were strictly restricted to those whose exclusion would introduce conceptual gaps or compromise the integrity of the clinical recommendations provided.

To provide an appropriate conceptual framework for interpreting the findings, an initial narrative review of foundational literature was conducted. This section summarizes well-established concepts regarding PC, sexuality, the clinical relevance of sexual health, and commonly described assessment and management approaches. These background sections are intended to contextualize the topic and should not be interpreted as findings derived from the 17 studies included in the evidence synthesis. 

What is palliative care?

According to the World Health Organization, PC is an approach that improves the quality of life of patients and their families who are facing problems associated with life-threatening illness [22]. This approach is carried out through an interdisciplinary team model, encompassing all dimensions of suffering, from physical, psychological, and social pain to family and spiritual needs [1], which in PC is known as total pain.

Ideally, this type of care should be introduced early following the diagnosis of any chronic illness, whether oncological or non-oncological, that threatens a person's quality of life. Furthermore, it is essential to emphasize that PC focuses not only on the patient but also on the entire family and caregivers [1]; therefore, it is imperative to assess their needs and provide support throughout the course of the illness.

One of the main ways to contribute to quality of life is through the early assessment and management of symptoms, including those that become refractory [22]. These interventions are not limited exclusively to hospital settings; they can also be provided on an outpatient basis or in the patient's home under the supervision of primary care physicians.

It is important to emphasize that PC provides holistic care to patients with life-limiting illnesses and should under no circumstances be considered an alternative reserved for failed life-prolonging treatments. On the contrary, its early implementation has been associated with improved survival in patients with metastatic cancer, reduced healthcare costs, and better quality of life [22].

What is sexuality?

To properly understand how chronic illness impacts the individual, it is important to establish the clinical definition of sexuality. The World Health Organization (WHO) defined sexuality in 2002 as, “A central aspect of being human throughout life encompasses sex, gender identities and roles, sexual orientation, eroticism, pleasure, intimacy, and reproduction. Sexuality is experienced and expressed in thoughts, fantasies, desires, beliefs, attitudes, values, behaviors, practices, roles, and relationships. While sexuality can include all of these dimensions, not all of them are always experienced or expressed. Sexuality is influenced by the interaction of biological, psychological, social, economic, political, cultural, legal, historical, religious, and spiritual factors” [22]. 

From a clinical assessment perspective, sexuality is addressed through three interconnected domains. The first is the biological domain, determined by physical capacity and the body's physiological response; the second is the psychological domain, centered on self-esteem, self-concept, and individual identity; and finally, the relational domain, based on emotional closeness and support within a partnership [23].

Current scientific evidence highlights that our sexuality changes throughout life. It is not fixed; rather, it adapts and transforms according to age, physical changes, and the health challenges faced by each individual [24]. 

Scoping the problem

Despite the established theoretical importance of holistic well-being, a profound disconnect exists between patient needs and actual clinical practice. Globally, an estimated 56.8 million people are in need of PC each year, of whom 25.7 million are in their last year of life [25]. Despite their condition, these patients retain the need for intimacy and sexual expression, the latter being a powerful form of expression that can help resolve personal matters [26].

Quantitative data from recent studies vividly illustrate the magnitude of this neglected domain. According to Bramati et al., who conducted a study with a sample of 100 PC patients, 81% reported experiencing sexual dysfunction in the last year, and 55% reported dissatisfaction with their sexual function. However, only 29% wished to discuss sexual dysfunction with a physician, but only 20% reported that their physician had ever asked them about sexual dysfunction. Furthermore, 79% considered it appropriate to be asked about their sexuality, while only 32% considered that it should always be a frequent question [12]. For their part, Kelemen et al. evaluated the impact of serious illnesses on the sexuality of PC patients; out of 97 patients, 91.7% reported that they had never been asked about their sexuality before, and 48.4% stated that their illness had significantly and negatively affected their intimacy [27]. Regarding sexuality in patients with advanced cancer, Vitrano et al. conducted a study with 65 patients, finding that 86% considered sexuality important enough to want to discuss it with a physician, and 47% reported that it was vital for their psychological well-being [28]. Finally, Rouanne et al. aimed to evaluate health-related quality of life and sexual function in patients with advanced cancer; in their study with 63 patients, 57% of women and 68% of men considered sexual quality of life to be an important factor [29].

Regarding the meaning of sexuality for PC patients, Lemieux et al. conducted interviews with 10 patients receiving care in a palliative environment. All participants felt that the impact of the illness on their sexuality should be addressed [30].

At least 21% of patients enrolled in PC programs express concern regarding a decrease in libido, with these figures being higher in specific populations [31]. For instance, between 56% and 80% of patients with amyotrophic lateral sclerosis or multiple sclerosis report libido issues [32]. Likewise, Hoekstra et al. conducted a study with more than 400 heart failure patients, in which 59% reported sexual dysfunction [33].

This pervasive neglect becomes even more acute when intersecting with advanced age. Population aging is an undeniable reality, with increasingly lower birth rates globally. In this context, some older adults gradually lose their autonomy as their frailty increases. This leads to them being considered asexual individuals, as it is assumed that age-related bodily changes diminish sexual interest. The de-sexualization of this age group constitutes a harmful restriction of sexual identity; furthermore, it has been suggested that this phenomenon is linked to the cultural association between old age and death [34].

It has been proposed that the sexuality of older individuals at the end of life within a PC setting may constitute a triple taboo: sexuality itself, old age, and death [34]. In this regard, Wallach et al. conducted a study among PC specialists working with older patients at the end of life. They demonstrated that the majority of physicians did not address sexual health with their patients, considering it inappropriate or irrelevant for their age, and even more so if the patients were single [35].

Despite the high incidence of sexual dissatisfaction among PC patients, only a minority are ever questioned about concerns associated with their sexuality [31]. A primary barrier is the lack of engagement by healthcare providers. As demonstrated by Bramati et al. [36], a stark contradiction exists in clinical practice: although PC specialists recognize the negative impact of sexual dysfunction on the quality of life of oncology patients, the majority omit routine assessment due to a lack of formal training, time constraints, and discomfort. This perpetuates a clinical silence that neglects an essential dimension of human beings during their final days.

Generally, healthcare personnel tend to view sexuality as a low priority within the context of PC, prioritizing symptom management such as pain, dyspnea, and nausea. This may stem from the fact that healthcare professionals do not feel equipped with the necessary tools to address this topic. In a study conducted by Kutner et al. involving 348 patients across 16 different hospices, healthcare providers reported feeling insufficiently informed or trained to assess issues related to patients' sexual activity [37].

Furthermore, clinicians often mistakenly infer that sexuality refers exclusively to genital-centered coitus. This narrow perspective invalidates other forms of sexual expression and connection that are not strictly genital-focused, such as massages, hugs, and physical touch. In patients who may be physically unable to engage in sexual intercourse due to their medical conditions, physicians often assume that sexuality is of little importance, which represents a significant oversight [9]. According to Gleeson and Hazell, a survey of healthcare professionals revealed that another critical reason for failing to address sexual well-being in oncology and PC patients was that it did not present as a chief complaint, or that providers were waiting for the patient to initiate the discussion [38]. Consequently, these findings demonstrate that healthcare professionals must take the initiative to address the issue proactively.

Impact of illness on sexuality

When evaluating the clinical reality of these patients, patient-reported outcomes from oncological populations in PC highlight a profound impact on sexuality; a recent survey indicated that up to 81% of these patients experienced sexual dysfunction and 55% reported dissatisfaction with their sexual function [12]. Regardless of patient biological sex, dysfunctional manifestations fall into two main categories: alterations in sexual response-such as loss of libido, erectile or ejaculatory dysfunction, hypo-lubrication, dyspareunia, and anorgasmia-and affective-cognitive disorders, characterized by a loss of motivation, diminished self-esteem, and a profound distortion of body image [11].

To systematically address these manifestations, clinicians must recognize that the causes of sexual health decline stem both from the disease itself and from the treatments administered. In patients undergoing PC, advanced chronic pathologies cause severe systemic symptoms such as chronic pain, refractory fatigue, and cachexia, triggering a high inflammatory burden and endocrine dysfunction that significantly reduces sexual desire [39]. Simultaneously, this biological problem is compounded by the routine introduction of essential palliative therapeutics, a classic example is opioid therapy, which suppresses the hypothalamic-pituitary-gonadal (HPG) axis, thereby inducing secondary hypogonadism that obliterates libido, causes erectile dysfunction in men, and induces severe vaginal dryness in women [40]. Psychotropic medications (antidepressants and neuroleptics) represent another heavily utilized drug class in PC that causes sexual dysfunction [41]. Furthermore, the use of exogenous glucocorticoids (such as dexamethasone, prednisone, or methylprednisolone) is a primary pharmacological cause of secondary hypogonadotropic hypogonadism in men through HPG axis suppression, affecting women through the exact same mechanism of action [42].

However, the degradation of intimacy in palliative settings is rarely a purely biochemical phenomenon; it is deeply reinforced by existential and psychological stress. The psychological impact of terminal illness profoundly alters the patient's self-perception and mental health. For instance, a study by Olsson et al. analyzed sexuality and self-perceived body image in patients with non-Hodgkin lymphoma. Their findings highlighted how drastic changes in appearance, such as massive weight loss, alopecia, and the presence of invasive medical devices (e.g., catheters, ostomies), leading individuals to perceive themselves as incompetent to engage in intimacy with a partner [43].

Beyond individual self-perception, terminal illness heavily reshapes interpersonal dynamics, the most critical role shift occurs within the couple, where the romantic partner abruptly transitions into the primary logistical caregiver in many cases. Compounding this, frequent hospitalizations and institutionalization deprive the couple of private spaces, effectively isolating the patient and eliminating opportunities for affective intimacy, skin to skin contact, or mutual support during the end of life stage [44]. 

Assessment tools

**Figure 1:**
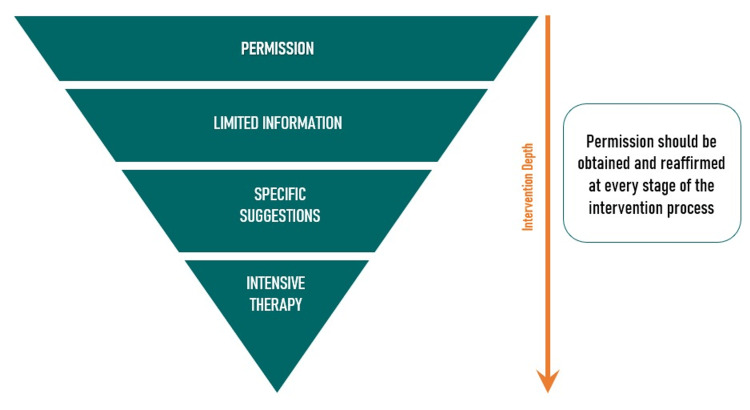
Modified PLISSIT model. Source: Adapted from Traverse et al. [26]. This figure was created using Microsoft PowerPoint (Microsoft Corporation, Redmond, WA). No artificial intelligence tools were used to create this figure. PLISSIT, Permission, Limited Information, Specific Suggestions, and Intensive Therapy

To address sexual health and sexuality comprehensively, the extended PLISSIT model can be utilized. This framework serves as a structured communication and intervention model designed to initiate discussion, provide clinical information, and guide patient referral in PC settings; its structure is described in Table 1. This model requires granting permission as a pivotal component across all phases, thereby empowering patients to decide how and to what extent to proceed. This approach is critical, as many healthcare professionals frequently bypass the permission-granting stage, proceeding directly to providing information without offering patients the opportunity to express their concerns. The objective efficacy of implementing this structured framework is well-supported by clinical data. A meta-analysis conducted by Bennett, which synthesized evidence from 15 intervention studies evaluating PLISSIT-based interventions for sexual dysfunction, reported a pooled effect size of Cohen's *d* = 1.00 (95% confidence interval (CI): 0.89-1.11). This corresponded to a Cohen's U3 value of 84%, indicating that approximately 84% of participants who received a PLISSIT-based intervention scored lower on measures of sexual dysfunction than the median participant in the comparison group, suggesting a substantial improvement in sexual functioning [14]. A brief training in healthcare professionals focused on needs of sexuality during end-of-life care facilitates comfort and ability to implement screening and interventions [26]. This model serves as a valuable decision-making tool, enabling clinicians to distinguish patients who can be managed effectively within primary care settings from those who require specialized intervention (Figure 1).

**Table 1:** Modified PLISSIT model. PLISSIT, Permission, Limited Information, Specific Suggestions, and Intensive Therapy

Stage	Description	Application
Permission (P)	Giving the patient permission to address their sexuality, while normalizing and validating their experiences	“The type of treatment you are receiving may affect your sex life. If you're comfortable with it, I would like to ask if you have any doubts or concerns about any changes you've noticed in your sexuality.”
Limited information (LI)	Providing objective and specific information on how the disease and treatment may be affecting sexuality, and correcting misconceptions	“Due to treatment side effects, changes in your sex life—such as pain during intercourse—are common. If you feel comfortable, I’d like to hear about your experience with these changes.”
Specific suggestions (SS)	Once the healthcare professional identifies a sexual dysfunction, they must provide specific advice on how to cope with the effects of the illness, including the use of pharmacological and non-pharmacological strategies.	Vaginal lubricants and moisturizers are effective in treating vaginal dryness and can help alleviate pain during intercourse. Try different sexual positions to reduce discomfort, or change the routine to have intimate relations when you have more energy—for example, in the morning instead of at night, planning the encounter in advance. To foster intimacy with your partner, practices that may be appropriate include focusing on touch, massages, caresses, and intimate communication instead of sexual intercourse.
Intensive therapy (IT)	In the case of clinical professionals who do not have the necessary competencies to address issues related to patients' sexual health in depth, it is advisable to refer them to a specialist to receive specialized supportive therapy.	“Your concern is entirely valid, and since it requires a specialized approach that goes beyond my area of expertise and practice, I believe it is appropriate to refer you to a professional who specializes in sexual health, who will be able to provide you with care tailored to your needs.”

In female patients, the SSCL for women after cancer can be utilized [15]. This is a brief screening tool designed to guide the identification of treatment-related sexual problems and to monitor symptoms over time. Its design allows for implementation regardless of marital status, sexual orientation, or current level of sexual activity. Although more comprehensive instruments are available to evaluate sexual function, they can be overly extensive and labor-intensive, thereby limiting their utility in routine clinical practice. For instance, the FSFI comprises 19 items assessing arousal, orgasm, satisfaction, and pain. Because administering all items is time-consuming, a validated six item abbreviated version of the FSFI has been developed [16]. Additionally, the PROMIS SexFS tool evaluates sexual function while offering greater inclusivity across diverse sexual orientations. However, its primary limitation resides in the fact that most evaluated parameters assume active sexual participation. This requirement may introduce information bias among female cancer survivors who experience severe sexual or vaginal health issues and have ceased sexual activity due to these symptoms [17].

Parallel to these female-specific indices, the assessment of male patients relies on tailored instruments that capture their distinct physiological challenges, the PROMIS SexFS, developed for men with cancer, can also be used; this tool consists of 8 questions evaluating erectile function, satisfaction, orgasm, and enjoyment. Alternatively, the IIEF is available, comprising 15 items that assess erectile function, orgasm, sexual desire, intercourse satisfaction, and overall satisfaction. Nevertheless, the IIEF presents limitations regarding ejaculatory evaluation, which cannot be accurately captured in patients undergoing radical prostatectomy, and its validation is primarily restricted to heterosexual men, thus limiting its applicability across different sexual orientations [17,18]. To address administration burden, an abbreviated 5-item version known as the IIEF-5 (or SHIM) is available to classify the severity of erectile dysfunction into five distinct categories [19]. Finally, the Expanded Prostate Cancer Index Composite (EPIC) is noteworthy for its capacity to evaluate sexual function and sexual bother; it consists of 20 disease-specific items for men with prostate cancer, including targeted subscales for hormonal, urinary, bowel, and sexual domains [20].

While the aforementioned tools provide deep insights into strictly gender-specific dysfunctions, the high prevalence of oncology patients in palliative settings frequently necessitates broader, bidirectional instruments. To this end, the EORTC has developed and validated a questionnaire specifically designed to evaluate quality of life and sexual health in cancer patients. This instrument, the EORTC QLQ-SH22, comprises 22 items distributed across four subdomains: desire, activity, satisfaction, and enjoyment, and includes specific items tailored for both men and women [21]. In clinical settings, its implementation facilitates the identification of the specific needs of patients and their partners regarding sexual difficulties, thereby optimizing the therapeutic approach by healthcare professionals [45].

Addressing sexuality

Comprehensive management of sexual health in PC does not aim to restore an idealized anatomical or reproductive function, but rather to optimize quality of life, preserve dignity, and adapt intimate well-being to the patient’s biological limitations.

Although there is no exclusive, single, standardized international clinical practice guideline specifically for addressing sexuality in PC, this gap in evidence-based medicine requires the multidisciplinary team to develop individualized therapeutic strategies. Healthcare providers have a responsibility to proactively initiate the conversation to break the taboo; when addressing sexuality in PC, the focus should be on non-coital emotional intimacy. The active application of the PLISSIT model is the recommended solution for overcoming the clinician’s barriers in day-to-day care [13].

To operationalize these strategies within the clinical domain, management should systematically begin by mitigating the primary biological and iatrogenic triggers of sexual decline, initially, a comprehensive review of the patient’s polypharmacy will be conducted to identify and, if symptomatic stability permits, rotate or reduce the doses of those medications with a harmful profile for the neuroendocrine axis, such as certain opioids or the chronic use of glucocorticoids [46]. In some cases, hormonal therapies may be considered, in women to address vaginal health, always under medical supervision and after analyzing contraindications [47]. 

The included studies consistently highlighted the importance of psychosocial support and effective communication in addressing body image concerns and relationship changes among patients receiving PC. Although specialist psychotherapeutic interventions have been recommended in the broader PC literature for couples experiencing significant relational distress, this recommendation was not directly evaluated within the studies included in this review. 

Psychosocial support is a central pillar for alleviating the affective-cognitive distress resulting from bodily disfigurement and changes in couple dynamics. While psychosocial support and effective communication should be addressed generally by all healthcare staff, in cases where the couple’s relationship has been substantially compromised, psychotherapeutic intervention by a trained specialist should be recommended [48]. 

Attention to body image can also help improve sexual health; in cases where the patient has drains, catheters, ostomies, or wounds, it is recommended to seek innovative solutions, with the support of trained staff, to perform these interventions [27]. 

Ultimately, because PC is inherently dynamic, these biomedical and psychosocial interventions cannot be treated as isolated events; they require systematic, longitudinal monitoring. Therefore, clinicians must monitor at each follow-up visit whether the implemented measures, such as medication changes, postural adjustments, or lifestyle modifications, have alleviated physical suffering, or if the natural progression of the disease requires an adjustment to comfort goals. Similarly, do not overlook the partner’s role as a caregiver; periodically assess the level of burden on the partner in their role as the primary logistical caregiver [48]. Follow-up care should provide therapeutic spaces to address frustration over the loss of the couple’s previous dynamic, offering communication tools that allow them to balance medical care with maintaining their emotional bond and mutual comfort until the end of life.

Synthesis of evaluated evidence

To synthesize the most relevant and representative scientific literature on this broad phenomenon, a comprehensive compilation of key studies was conducted. The main findings from the 17 articles analyzed, which reflect the perceptions of patients, partners, and HCPs regarding sexuality in PC. The outcomes of the articles were classified based on five specific parameters: (1) Impact of the disease on sexuality, 2) prevalence of sexuality assessment or discussion, (3) which assessment tools were used, (4) barriers and limitations to addressing the topic, and (5) interventions/suggestions. To ensure clarity and synthesis, the 17 studies were categorized into three distinct thematic groups: patient perspectives and experiences (Table 2), perspectives and barriers among healthcare professionals (Table 3), and literature reviews and conceptual frameworks (Table 4).

**Table 2:** Patient perspectives and experiences. *n*, number of participants; HCP, healthcare professionals; N/A, not applicable; N/E, not explicitly reported; D, diagnoses; NOS, Newcastle-Ottawa Scale; OCEBM, Oxford Centre for Evidence-Based Medicine; CASP, Critical Appraisal Skills Program; COREQ, Consolidated Criteria for Reporting Qualitative Research; AXIS, Appraisal Tool for Cross-Sectional Studies; χ², chi-square test; SD, standard deviation; PLISSIT, Permission, Limited Information, Specific Suggestions, and Intensive Therapy; EORTC QLQ-SH, European Organisation for Research and Treatment of Cancer Quality of Life Questionnaire Sexual Health; BIS, Body Image Scale; ADT, androgen deprivation therapy; ECOG, Eastern Cooperative Oncology Group; GPS, good performance status; PPS, poor performance status; PRISMA-ScR, Preferred Reporting Items for Systematic Reviews and Meta-Analyses Extension for Scoping Reviews; PC, palliative care; LVAD, left ventricular assist device; HIV/AIDS, Human Immunodeficiency Virus/Acquired Immunodeficiency Syndrome

Author/Year	Research design	Population characteristics	Study quality (SQ)/Level of evidence (LE)/Risk of bias (RB)	Limitations (research-focused)	Outcome measured
van der Meer et al. (2026) [21]	Prospective, multicenter, national observational cohort study (designated as the eQuiPe study). Use of questionnaires. Between 2017 and 2020.	*n* = 352 patients and their partners. Age range: 55-66 years. Place: 40 hospitals in the Netherlands. D: Lung cancer, colorectal cancer, breast cancer, prostate cancer.	SQ, 7/9 (NOS), multicenter study, clear criteria, not all factors controlled; LE, II (OCEBM), prospective cohort; RB, Moderate (ROBINS-I), risk of bias due to loss to follow-up and self-reporting bias	Small effective sample sizes, self-reporting, lack of cultural diversity, and limited longitudinal follow-up	(1) Sexual desire decreased significantly toward the end of life. Patients with poorer physical function and prostate cancer reported a greater decline. (2) Most patients and their partners indicated that HCPs did not systematically address sexuality during care. (3) EORTC QLQ-SH22 is a validated questionnaire for sexual health in oncology. (4) Discussing sexuality in an end-of-life context remains difficult for both patients and professionals. (5) Use of brief PROMs such as the EORTC QLQ SH22 to facilitate clinical conversation. Integrating sexual health into routine care and training HCPs is essential.
Kelemen et al. (2019) [27]	Mixed-methods study (quantitative with descriptive statistics and chi-square analysis, and qualitative with constant comparative methodology). Questionnaires.	*n* = 97 hospitalized patients. Age range: 18-80 years (mean, 57.9 years); African American, 71.1%. Place: hospitals with PC in the United States. D: cancer (43.3%), advanced heart failure (18.6%), advanced lung disease (8.2%), sepsis/infection (5.2%), other (21.6%).	SQ: 6-7/9 (NOS), moderate-good, multicenter study, clear criteria. LE: IIb (OCEBM), prospective cohort, qualitative and quantitative results. RB: Moderate (ROBINS-I), risk of self-reporting bias, lack of adjustment for covariates, and cultural bias.	Moderate sample size, limited generalizability, risk of self-reporting	(1) 48.4% reported that the illness moderately or significantly affected their intimacy, particularly among younger patients and those with partners. (2) 91.7% had never been asked about intimacy before their PC consultation; 81% found the conversation helpful. (3) Clinical questionnaires and qualitative interviews were used. (4) Lack of professional initiative, fear of being intrusive, and insufficient training; older patients or those without partners showed less interest. (5) Incorporate routine intimacy screening into consultations, train professionals, and recognize that intimacy includes both sexuality and affection.
Bramati et al. (2025) [12]	A cross-sectional study was conducted through a descriptive survey	*n* = 100 patients. Age range: ≥18 years; African American, 71.1%. Place: Outpatient Supportive Care Clinic, TX. D: advanced cancer in a palliative context.	SQ: 5-6/9 (NOS), moderate, no validated instrument, no possible causality. LE: III (OCEBM), cross-sectional study. RB: Moderate (ROBINS-I), self-reporting bias, lack of adjustment for covariates.	Self-report bias, limited generalizability, lack of validated tools, and limited scope focused on the patient perspective	(1) 81% reported sexual dysfunction in the past year; 55% were dissatisfied with their sexual function. (2) Only 20% said doctors asked about it; 91% had never been asked before. (3) No validated tools were used; only a self-administered survey. (4) Barriers included lack of physician initiative, fear of being intrusive, and limited training; only 32% thought it should always be asked. (5) Recommendation: adopt a flexible, patient-sensitive approach; recognize that many patients find it appropriate to be asked, but tailor discussions to individual preferences.
Wallach et al. (2026) [49]	Interpretive qualitative design based on individual semi-structured interviews and guided by an adapted reflexive thematic analysis.	*n* = 10 older adults. Age range: 69-86 years (mean, 76; 1 unknown). Place: three PC programs in Québec, Canada (hospital units, in-home programs, and hospice day centers). D: advanced or terminal stages of incurable diseases (lung, colorectal, vaginal, kidney, and unspecified cancers, and Parkinson's disease).	SQ: Moderate (CASP qualitative) due to a small sample size and a broadened recruitment strategy prompted by field difficulties and pandemic restrictions. LE: V (OCEBM level of evidence for qualitative studies). RB: Moderate to high (based on NOS domains); inherent self-reporting bias, selection bias due to the highly taboo and voluntary nature of the topic, and reflexivity bias (mitigated through research team dialogue).	Small sample size due to the pandemic and recruitment taboos, which limited full data saturation. 100% Caucasian Canadians and 90% heterosexual participants, heavily limiting transferability to diverse demographic groups.	1(1) High physical and psychological impact. Symptoms (dyspnea, weakness) and treatments (ostomy bags) diminish desire or stop genital activities, leading to relational distancing and internalized ageist/ableist self-exclusion. (2) The theme is highly taboo and rarely addressed in the literature or clinical practice. Healthcare professionals experienced evident discomfort approaching potential participants. (3) N/A. (4) Intersecting taboos around old age, severe illness, and mortality. Ageist/ableist biases from clinicians, partners, and patients. Restrictions and staff constraints during the COVID-19 pandemic. (5) Providing professional support to process the loss of physical capacity. Deconstructing clinical biases. Legitimizing internalized modes of expression (fantasies, dreams, memories) to support identity and well-being
Hay et al. (2020) [50]	Cross-sectional survey study (sub-analysis of a larger descriptive study)	*n* = 83 gynecologic cancer outpatients. Age range: 32-87 years. Place: Gynecologic Oncology Clinic at a large academic medical center (Magee-Womens Hospital of the University of Pittsburgh Medical Center), United States. D: current or prior gynecologic cancers, predominantly ovarian cancer (36%) and uterine/endometrial cancer (29%), followed by cervical, vulvar, and vaginal cancers.	SQ: 6/9 (NOS), moderate to high, showing clear selection criteria, defined outcomes, and appropriate statistical tests (χ² and Fisher exact tests), though it lacks long-term follow-up. LE: IIb (OCEBM). This satisfies the criteria for a cross-sectional observational study. RB: Moderate to High (NOS/AXIS adapted). Potential for self-reporting bias inherent to anonymous surveys and selection bias due to convenience sampling at a single institution.	The study included a relatively small number of patients (*n* = 83) at a single academic institution, which limits its generalizability to other environments. Furthermore, the patient population was predominantly White (87%), meaning the findings may not be generalizable to other racial or ethnic groups.	(1) Gynecologic cancers and their respective medical treatments directly disrupt sexuality, intimacy, and overall sexual function across a broad range of patient ages and cultures. (2) Exactly 57% of the surveyed patients reported that sexuality and intimacy were never discussed at any point during their oncologic cancer care. (3) Data were gathered using an anonymous, cross-sectional, investigator-developed survey explicitly evaluating experiences, preferences, and barriers. (4) The primary barrier identified was patient discomfort (reported by 28% of patients). Minor barriers included a lack of provider time (5%), provider discomfort (4%), and structural provider factors, such as a lack of specialized training or resources (<2%). (5) The authors recommend initiating conversations early to address key issues related to maintaining sexual function (e.g., the use of vaginal dilators in patients undergoing radiation therapy) and diving into broader psychosexual topics during follow-up. They also suggest using pre-visit questionnaires to screen for relationship status and sexual activity, implementing the PLISSIT model, and normalizing discussions to actively reduce patient discomfort.
Schmalz et al. (2024) [45]	Observational cohort study (secondary analysis of the international validation of the EORTC QLQ SH22).	*n* = 101 patients. Age range: 20-80 years (76.8% with a partner). Divided by functional status into 2 subgroups: GPS group (*n* = 66) and PPS group (*n* = 32). Place: multiple European and Asian hospitals within the EORTC network. D: breast (14.9%), gynecological (12.9%), prostate (10.9%), lung (17.8%), colorectal (10.9%), and head and neck (9.9%) tumors.	SQ: 7/9 (NOS), moderate-good, multicenter study, clear criteria, not all factors controlled. LE: IIb (OCEBM), prospective cohort (not randomized). RB: Moderate (ROBINS-I), risk of self-reporting bias and lack of adjustment for covariates.	Cultural and clinical heterogeneity. Risk of self-report bias, lack of adjustment for covariates, and lack of representativeness (only hospitalized cancer patients).	(1) PPS reported lower sexual satisfaction, more fatigue, and lower libido. GPS reported greater effects of treatment on sexual activity. (2) Communication with healthcare professionals was very low in both groups (GPS, 6.5%; PPS, 14%). (3) EORTC QLQ-SH22 (validated PROM). (4) Low communication with healthcare professionals. Lack of training for professionals to address sexuality. (5) Clinical use of the EORTC QLQ-SH22 to facilitate conversations, along with professional training and practical privacy and communication measures.
Kislev et al. (2022) [51]	Qualitative research based on interviews (analyzed using the constant comparative method).	*n* = 35 patients. Age range: N/E. Place: hospitals and cancer centers linked to the Israel Cancer Association. D: incurable cancer or stage of dying from cancer	SQ: Moderate (CASP), small sample, no age range or detailed sociodemographic characteristics. LE: III (OCEBM), descriptive qualitative/observational study. RB: Moderate high (ROBINS-I), small sample, risk of cultural bias (article in Hebrew).	Small sample size, cultural and geographical bias, risk of self-reporting, and qualitative study design.	(1) Many patients reported a disappearance or decrease in their sexuality; some maintained satisfactory intimacy, with varying levels of discomfort. (2) Most considered it important to address the topic; one-third expressed a need for sexual counseling. (3) Only qualitative interviews, without standardized instruments. (4) Sexuality and death are taboo subjects, which makes them difficult to address; some patients wish to talk about the topic, while others do not. (5) It is recommended that the PC team initiate the conversation, recognize the importance of intimacy even in the terminal phase, and offer sexual counseling to those who request it.
Janecki et al. (2021) [52]	Prospective pilot, multicenter, observational study, conducted using EuroQoL EQ-5D-3L and a self-developed questionnaire.	*n* = 342 patients (60% women, 40% men). Age range: 18-80 years (61 79 years, 70.3%). Place: home-based PC programs in Poland. D: digestive neoplasms (24.3%), breast cancer (17.3%), respiratory neoplasms (16.7%), other genitourinary and hematological tumors; only 3.5% non-cancerous.	SQ: 6-7/9 (NOS), moderate-good, multicenter study, use of a validated instrument (EQ-5D-3LLE: IIb (OCEBM), prospective cohort observational. RB: Moderate (ROBINS-I), risk of self-reporting bias and cultural bias, use of a questionnaire not validated (developed by authors).	Risk of self-reporting, limited generalization (Polish population, homebound)	(1) 56.9% reported a worsening of their sex life after diagnosis, especially men (71.7%). (2) 47.4% felt that the PC team did not acknowledge their sexual problems; only 4.5% received explicit help. (3) The validated EQ-unvalidated questionnaire was used. (4) Lack of standardized tools in Polish, cultural taboos, time constraints, and lack of training and confidence among professionals. (5) It is recommended to train physicians and nurses in communicating about sexuality, validate simple instruments, use the EQ-5D-3L as a support tool, integrate assessment into routine practice, and refer patients to sexology services when necessary.
Kelemen et al. (2016) [53]	A pilot study using a mixed methods design to evaluate a screening tool.	*n* = 57 inpatients (60% women, 40% men), African American (68%). Age range: mean 58 years (SD 14.4). Place: 2 hospitals in the United States. D: heart failure (84.2%), cancer (10.5%), other (5.3%).	SQ: 5-6/9 (NOS), moderate, small sample size, study using an easy-to-use instrument, but not validated. LE: III (OCEBM), descriptive/pilot cross-sectional study. RB: Moderate (ROBINS-I), risk of self-reporting bias, lack of adjustment for covariates, and cultural bias.	Pilot study lacking statistical power, small sample size. Tool not internationally validated. Cultural and hospital bias (African American patients, only 2 urban centers).	(1) 56.2% reported that the illness significantly or moderately impacted their intimacy. In end-of-life patients, 70.5% reported a significant/moderate impact. (2) 96% had never been asked about intimacy before. All end-of-life patients found the discussion helpful. (3) Brief screening tool not validated, but practical for clinical use. (4) Lack of privacy in hospitals. Insufficient training of professionals to address intimacy. Erroneous assumptions based on age, diagnosis, or functional status. (5) Incorporate simple questions about intimacy into routine PC consultations. Train professionals to address the topic sensitively. Consider using written forms to facilitate openness on sensitive issues.
Kelemen et al. (2022) [54]	A descriptive qualitative study based on semi-structured interviews and thematic analysis.	*n* = 21 hospitalized patients (men 66%, women 33%). Age range: mean 63 years (SD 14), African American (86%). Place: one hospital in Washington, USA. D: heart failure (12), cancer (4), HIV/AIDS (2), lung disease (2), vascular disease (1).	SQ: Moderate-high (CASP qualitative), clear criteria, analytical rigor, but limited representativeness. LE: III (OCEBM), exploratory qualitative study. RB: Moderate high (ROBINS-I), risk of self-reporting bias, unique context, small sample, cultural bias.	Small sample size. Dominant diagnosis: heart failure. Possible cultural and religious bias (African American patients and Christians).	(1) 76% reported an impact on sexual activity due to physical changes and treatment; relationships adapted, some even strengthened. (2) 76% said no doctor had discussed intimacy; communication was limited to sexual restrictions. (3) No validated tools were used, only semi-structured interviews. (4) Barriers included lack of physician initiative, shame, cultural taboos, and prioritization of illness over intimacy. (5) Address intimacy as part of PC, broaden its definition to include affection and emotional support, and consider faith/spirituality as resilience.

**Table 3:** Healthcare professionals' perspectives and barriers. n, number of participants; HCP, healthcare professionals; N/A, not applicable; N/E, not explicitly reported; D, diagnoses; NOS, Newcastle-Ottawa Scale; OCEBM, Oxford Centre for Evidence-Based Medicine; CASP, Critical Appraisal Skills Program; COREQ, Consolidated Criteria for Reporting Qualitative Research; AXIS, Appraisal Tool for Cross-Sectional Studies; χ², chi-square test; SD, standard deviation; PLISSIT, Permission, Limited Information, Specific Suggestions, and Intensive Therapy; EORTC QLQ-SH, European Organisation for Research and Treatment of Cancer Quality of Life Questionnaire Sexual Health; BIS, Body Image Scale; ADT, androgen deprivation therapy; ECOG, Eastern Cooperative Oncology Group; GPS, good performance status; PPS, poor performance status; PRISMA-ScR, Preferred Reporting Items for Systematic Reviews and Meta-Analyses Extension for Scoping Reviews; PC, palliative care; LVAD, left ventricular assist device; HIV/AIDS, Human Immunodeficiency Virus/Acquired Immunodeficiency Syndrome

Author/Year	Research design	Population characteristics	Study quality (SQ)/Level of evidence (LE)/Risk of bias (RB)	Limitations (research-focused)	Outcome measured
Wallach et al. (2023) [35]	Interpretive qualitative design based on individual semi-structured interviews, guided by reflexive thematic analysis.	*n* = 16 PC professionals (13 women, 3 men), including nurses (*n* = 6), physicians (*n* = 3), social workers (*n* = 3), and other therapists/assistants. Age range: professionals aged 25-65 years. Place: interdisciplinary PC programs (hospital and home care settings) in Montreal, Canada. D: N/E	SQ: Moderate to High (CASP qualitative) for qualitative standards due to rigorous thematic methodology, data triangulation, and peer debriefing/member checking with head nurses. LE: Level V (OCEBM), evidence from an individual descriptive or qualitative study. RB: Moderate risk (based on NOS domains) of selection bias due to voluntary participation and a small sample drawn from a single institutional network.	Small sample size, comprising only 16 professionals from one regional health network, which limits the transferability of the findings to broader PC contexts. Missing patient voices; the study relies exclusively on the observations, perceptions, and potential biases of healthcare staff, completely omitting the direct lived experiences and perspectives of the older adults themselves.	(1) Severe physical decline, fatigue, and weakness associated with dying are perceived by professionals to heavily drain energy, shifting patient expression away from genitality and primarily toward non-genital touch, tenderness, and emotional connection. (2) Very low. The vast majority of care providers admit they never proactively initiate conversations about or evaluate sexuality with older patients. (3) N/A. (4) Driven by a triple taboo of aging, sexuality, and death. Rooted in deep ageist assumptions (associating old age with asexuality), ableist biases (viewing weak/dying bodies as incapable of sex), heterosexist framing, personal discomfort, lack of formal clinical training, and pandemic workload strain. (5) Implementing targeted professional training on older adult end-of-life sexuality, routinely assessing intimacy needs during care planning, and integrating sexologists into clinical referral pipelines.
Bramati et al. (2023) [36]	Quantitative, cross-sectional descriptive study (pilot exploratory survey) using a voluntary, anonymous electronic questionnaire administered via Qualtrics software.	*n* = 49 PC clinicians, including physicians, advanced practice providers, and counselors/psychologists. Age range: not specified. Place: Department of PC, Rehabilitation and Integrative Medicine at The University of Texas MD Anderson Cancer Center in Houston, TX. D: N/E.	SQ: Moderate-Good. 6 7/9 (NOS) adapted for cross-sectional studies. Strengths include a high response rate (89%) and a multidisciplinary consensus blueprint for the questionnaire, though it lacks psychometric validation due to its pilot nature. LE: Level VI (OCEBM) (evidence from a single descriptive or cross-sectional study). RB: Moderate risk (based on NOS domains). Potential for social desirability bias given the sensitive nature of the topic. Risk of selection bias due to voluntary participation and limited generalizability as a single-center study.	Small single-center sample, because it is a small convenience sample from a single tertiary specialized institution (MD Anderson), the findings reflect local practice patterns and limit broader generalization. Exclusion of deep demographic variables, to strictly guarantee and protect participant anonymity within a small staff pool, specific background characteristics such as country of birth, ethnic group, or religion could not be collected. Social desirability and selection bias, discussing sexual dysfunction can carry an inherent taboo, which may lead participants to answer according to social expectations or cause selective bias among those who agreed to complete the survey.	(1) The literature review notes that sexual dysfunction (SD) affects 40% to 100% of oncology patients and remains highly significant even at the end of life. (2) Very low. 69% (*n* = 34) of clinicians reported that they rarely or never discuss sexuality with their patients. Furthermore, 96% (*n* = 47) stated that patients brought up SD only half of the time or never. However, 80% (n 39) will discuss it when asked by the patient. (3) None in practice. 86% (*n* = 42) of respondents had never heard of the PLISSIT model. 73% (*n* = 36) never or only half of the time discuss the side effects of medications on sexuality. (4) The top reasons for not discussing SD were: the patient did not raise the issue (76%), lack of time (71%), the presence of a third party (49%), and the patient being too ill (43%). Additionally, 59% (*n* = 29) believe it is the medical oncologist's responsibility. (5) 86% agree it is important to screen for SD. 84% acknowledge the need to receive more training, and 82% feel it is important to increase their knowledge. 67% agree that providing printed materials (brochures, pamphlets) would be helpful. 59% admitted they do not know how to access information regarding SD.
Benoot et al. (2018) [55]	Generic qualitative study. Based on in-depth interviews, thematic analysis was used to analyze the data.	*n* = 21 nurses who work in inpatient and outpatient PC. Age range: 28-58 years. Place: PC networks in Flanders, Belgium. D: N/E.	SQ: Moderate to High (CASP). LE: III (OCEBM). This corresponds to a qualitative/descriptive study. RB: Moderate (CASP)/transparent reporting (COREQ). Potential self-selection/motivation bias exists since recruitment relied heavily on the nurses’ willingness and motivation to discuss a sensitive topic like sexuality.	Limited transferability due to a small, motivation-biased sample restricted entirely to the healthcare infrastructure of Flanders, Belgium. The study excluded nurses working in other areas, such as the oncology department.	(1) The PC stage brings about profound changes in this dimension, as physical deterioration progresses (immobility, general malaise), diminishes desire, and the approach of death triggers intense emotional reactions that either strengthen the bond or cause relational withdrawal due to a lack of communication. (2) N/A; however, the text notes that nurses often avoid or give lower priority to the topic of sexuality in their daily practice. (3) Data collection relied strictly on qualitative, semi-structured in-depth interviews guided by a broad conceptualization of intimacy. (4) Obstacles include environmental limitations (lack of privacy), nurses’ emotional barriers (fear, embarrassment, feeling unqualified), care that focuses on the disease rather than the person, which leads to prioritizing physical symptoms while neglecting sexuality, and taboos regarding the asexuality of the sick or elderly. (5) The authors suggest a “sex-positive approach” through interpersonal strategies like active listening and open probing, negotiating routines, adapting infrastructure for privacy, and utilizing team reflection to overcome professional discomfort.
Hjalmarsson et al. (2020) [56]	A descriptive qualitative study with an interpretive approach and qualitative analysis.	*n* = 11 nurses who work in specialized inpatient and outpatient PC units. Age range: 25-60 years old. Place: three specialized public/county-run PC units in southern Sweden. D: N/E	SQ: Moderate to High (CASP). LE: III (OCEBM). This satisfies the criteria for descriptive or qualitative studies. RB: Moderate (CASP). There is a potential for self-selection or motivation bias, since the participants volunteered to take part after receiving information about a sensitive and taboo research topic.	The transferability of the findings is limited to similar contexts within a specific region in the south of Sweden or countries operating under identical palliative policies; furthermore, the study intentionally excluded nurses working with PC in other care units (e.g., regular hospital wards).	(1) Advanced physical deterioration (fatigue, pain, ostomies, odors) triggers bodily shame, while fear of injury causes couples to shift their sexual expressions away from intercourse toward physical closeness and touch. (2) N/A; however, the study notes that explicitly addressing sexual health needs or patient expressions is extremely rare in daily practice. (3) Data collection was strictly conducted through three semi-structured focus group interviews. (4) Obstacles include institutional deficits (lack of protocols, checklists), nurses’ personal embarrassment, ward privacy limitations, and personal biases regarding age and gender. (5) Recommendations include integrating sexuality into formal nursing curricula, incorporating explicit questions into routine checklists, providing educational brochures, promoting tactile massage/skin contact, and utilizing managerial leadership to enforce guidelines.
Gleeson et al. (2017) [38]	Quantitative, cross-sectional descriptive study utilizing an anonymous electronic questionnaire deployed via SurveyMonkey.	*n* = 121 healthcare professionals. Professional groups consisted of nurses (59%), doctors (33%), healthcare support workers (4%), surgeons (3%), and allied health professionals (1%). Age range: N/A (not reported for the staff). Place: One regional Health Board in Wales, UK. Participants were drawn from primary care (district nurses, GPs) and secondary care (oncology and specialist PC teams). D: N/E.	SQ: Moderate-Good. 6 7/9 (NOS) adapted for cross-sectional studies. LE: Level VI (OCEBM), evidence from a single descriptive or cross-sectional study. RB: Moderate Risk (based on NOS domains) (Risk of self-reporting/social desirability bias where staff might overreport favorable practice; significant non-response bias due to low participation among district nurses (8%) and GPs (40%)).	The total sample is relatively small, with very low representation from surgeons and allied health professionals, limiting generalizability. Low response rates in primary care: while PC staff had a 100% response rate, district nurses (8%) and GPs (40%) had low participation. Potential self-reporting bias: Relying on a voluntary survey format introduces a risk of positive self-reporting bias.	(1) The study notes that cancer and its treatments cause persistent, distressing sexual dysfunction in 20% to 100% of patients, heavily compromising long-term sexual well-being. (2) The majority of staff do not routinely assess sexual well-being (80% of GPs, 73.7% of district nurses, and 65.5% of secondary care staff do not routinely check). A subgroup fails to assess it at all (8% of GPs, 13% of district nurses, 13.8% of secondary care). Only 13.8% of secondary care, 7.9% of district nurses, and 4% of GPs routinely assess it. (3) Extremely Low. The overwhelming majority of primary care staff and 80% of secondary care staff do not use any clinical tools to aid in the assessment of sexual well-being. (4) The primary barrier was that sexual well-being was "not the presenting problem" during consultations. Other highly endorsed barriers include waiting for the patient to raise it, not being the focus of the appointment, lack of an appropriate physical setting, perception of it as an invasion of privacy, and lack of professional training. A minority of GPs (12%) and district nurses (10.5%) felt it was not a legitimate clinical issue. (5) Respondents heavily endorsed a need for training and support to facilitate routine assessment. Key requests included better knowledge of specialist services for patient referrals, access to patient-facing written educational resources, and standard clinical assessment tools or guidance.

**Table 4:** Literature reviews and conceptual frameworks. n, number of participants; HCP, healthcare professionals; N/A, not applicable; N/E, not explicitly reported; D, diagnoses; NOS, Newcastle-Ottawa Scale; OCEBM, Oxford Centre for Evidence-Based Medicine; CASP, Critical Appraisal Skills Program; COREQ, Consolidated Criteria for Reporting Qualitative Research; AXIS, Appraisal Tool for Cross-Sectional Studies; χ², chi-square test; SD, standard deviation; PLISSIT, Permission, Limited Information, Specific Suggestions, and Intensive Therapy; EORTC QLQ-SH, European Organisation for Research and Treatment of Cancer Quality of Life Questionnaire Sexual Health; BIS, Body Image Scale; ADT, androgen deprivation therapy; ECOG, Eastern Cooperative Oncology Group; GPS, good performance status; PPS, poor performance status; PRISMA-ScR, Preferred Reporting Items for Systematic Reviews and Meta-Analyses Extension for Scoping Reviews; PC, palliative care; LVAD, left ventricular assist device; HIV/AIDS, Human Immunodeficiency Virus/Acquired Immunodeficiency Syndrome

Author/Year	Research design	Population characteristics	Study quality (SQ)/Level of evidence (LE)/Risk of bias (RB)	Limitations (research-focused)	Outcome measured
Traverse et al. (2025) [26]	An exploratory literature review, a scoping review, was used according to PRISMA-ScR guidelines. 18 reviewed articles.	*n* = > 700 patients (some with partners), 220 caregivers, > 200 HCP. Age range: 12-84 years (adolescents to older adults). Place: Hospitals, hospices, oncology centers, residential care facilities, and community surveys are most common in developed countries. D: Wide spectrum: cancers (breast, lung, GI, pancreatic, prostate), heart failure, hepatocellular carcinoma, motor neuron disease, and non-malignant life-limiting conditions.	SQ: Moderate to high (PRISMA-ScR compliant) LE: V (OCEBM) Scoping Review RB: Moderate (ROBINS-I), no risk-of-bias assessment, small samples, self-report, and provider bias.	Heterogeneity, small samples, more qualitative studies, possible cultural and geographical bias (most health systems are in developed countries), and lack of systematic quality assessment.	(1) Sexual satisfaction and activity decline sharply (Vitrano: 83.3% → 12.1%). Sexuality is a frequent symptom in hepatocellular carcinoma (Hansen). Between 50% and 76% of patients report intimacy problems. (2) 92% of patients were never asked about sexuality (Kelemen). Professionals rarely address it (Gleeson). Even when 80% discussed dysfunction, most admitted lacking training (Bramati). (3) The PLISSIT model reduced dysfunction by 84%, yet 86% of clinicians were unfamiliar with it (Bramati et al.). (4) Provider discomfort, insufficient training, personal values, privacy issues, and cultural taboos. In Indonesia, sexuality is considered inappropriate to discuss (Purba). (5) Innovative and person-centered interventions (e.g., music therapy, expansive definitions of intimacy, privacy measures, LGBTQIA+ inclusivity) show promise in improving patient and partner well-being.
Wang et al. (2018) [57]	Narrative review (literature review based on Medline database searches). 11 reviewed articles.	*n* = 300 approximately. Age range: 12-83 years (adolescents to older adults), 18-80 years (mean 57.9 years). Place: cancer hospitals, PC units, hospices, hepatology clinics, pediatric clinics, and online surveys in developed countries. D: Predominantly cancer (breast, lung, gastrointestinal, urogenital, hematological, etc.), heart failure, other life-limiting diseases, non-malignant (e.g., motor neuron disease).	SQ: Low (AMSTAR2). While the review presents extensive research and clear inclusion criteria, it receives a low rating because it lacks a formal quality evaluation of the individual studies included. LE: V (OCEBM) narrative review/expert opinion (without meta-analysis) RB: Moderate-high (adapted ROBINS-I), risk of self-reporting bias, lack of adjustment of covariates, selection bias.	Small sample sizes, a narrative study with compilation of more qualitative and descriptive studies, fewer validated tools, self-report bias, and selection bias.	(1) Sexuality remains important for terminally ill patients, though symptoms and treatments markedly reduce satisfaction and activity. (2) Discussions about sexuality in PC are reported to be infrequent. For example, in adolescents, no physician took a sexual history; in adults, most were never asked. (3) Frameworks such as PLISSIT, BETTER, 5As, ALARM, and Stepped Skills are available, but no universal gold standard exists. (4) Professional limitations include lack of training, discomfort, time constraints, and assumptions about age or condition (e.g., adolescents or the elderly not considered sexually active). (5) Formal sexual health training, integration of psychosexual support, structured models, and referral to specialists are recommended.

The final synthesis included 17 studies published between 2016 and 2026; nearly all studies were conducted in high-income countries, particularly the United States, Canada, the United Kingdom, the Netherlands, Sweden, Belgium, Poland, and Australia, highlighting the limited evidence available from low- and middle-income settings [26]. Regarding study populations, most research focused on adults and older adults receiving PC, with mean or median ages generally exceeding 55 years. Only a few studies included younger populations, covering young adults and adolescents [26,27,45,52,57]. Advanced cancer was the most frequently investigated condition, although several studies also included patients with advanced heart failure, hepatocellular carcinoma, HIV/AIDS, neurodegenerative disorders, and other life-limiting illnesses [12,21,26,27,45].

The included studies demonstrated considerable methodological heterogeneity, although several common characteristics were identified. Most investigations employed observational designs, including prospective cohorts, cross-sectional surveys, and qualitative studies, while only two literature reviews were included [26,57], and no interventional studies were identified, limiting the evidence to recommendations and tools to practical implementation. Methodological quality was predominantly moderate to moderate-high, with most studies providing Level II-III evidence according to the Oxford Centre for Evidence-Based Medicine classification. Nevertheless, nearly all studies presented a moderate risk of bias, mainly attributable to self-reported outcomes, relatively small sample sizes, convenience sampling, and limited adjustment for potential confounding variables.

Impact of Disease on Sexuality

Across all included studies, advanced illness was consistently associated with substantial changes in patients' sexuality and intimate relationships. Progressive physical deterioration-including fatigue, pain, dyspnea, weakness, body image changes, and treatment-related adverse effects-contributed to reduced sexual desire, decreased sexual activity, impaired sexual satisfaction, and increased sexual dysfunction. Several studies reported that approximately 50% to 75% of patients experienced moderate-to-severe impairment of intimacy, while others demonstrated marked reductions in sexual activity as disease progressed [12,21,26,27,45,52,53,54].

Beyond genital sexual activity, several qualitative studies described a gradual shift toward broader expressions of intimacy. Couples frequently adapted their relationships by emphasizing affection, physical closeness, emotional support, cuddling, and other non-genital forms of intimacy [26,27,35,49,54]. In some cases, these adaptations strengthened interpersonal relationships, whereas others experienced distancing due to physical limitations, emotional distress, or communication difficulties [35,49,51,54,55].

Prevalence of Sexuality Assessment or Discussion

Sexuality remained consistently underassessed throughout PC services. Across multiple studies, the majority of patients reported that HCP had never initiated conversations regarding sexuality or intimacy, with proportions frequently exceeding 75% [26,57] and reaching more than 90% in several investigations [12,27,53,54]. Similar findings emerged from studies involving HCP, who acknowledged that routine assessment of sexual concerns was uncommon in both hospital- and community-based PC settings [35,36,38,55,56].

Despite this limited clinical practice, patients generally perceived these discussions positively when they occurred [28,36]. Several studies found that conversations about sexuality were considered appropriate, useful, and beneficial, suggesting a substantial mismatch between patients' expectations and routine professional practice [26,27,51,57].

Assessment Tools

Considerable heterogeneity was observed in the instruments used to evaluate sexuality. Most quantitative studies relied on investigator-developed questionnaires, whereas qualitative studies employed semi-structured interviews, reflecting the absence of standardized assessment strategies in PC.

Among validated instruments, the EORTC QLQ-SH22 was the most frequently reported questionnaire, particularly in patients with advanced cancer [21,26,45]. Several publications also highlighted structured communication models, including PLISSIT, BETTER, 5As, ALARM, and Stepped Skills, as potentially useful frameworks for integrating sexual health assessment into routine care [26,57]. However, these models were rarely implemented in clinical practice, and many HCP reported limited familiarity with them [12,36,38].

Barriers and Limitations

Several recurring barriers limited the routine assessment of sexuality in PC. The most commonly reported obstacles included insufficient professional training, lack of confidence, personal discomfort, limited consultation time, inadequate privacy, and prioritization of physical symptoms over psychosocial concerns [27,35,36,50,52,53,57]. HCP also frequently reported waiting for patients to raise the topic, whereas patients often expected clinicians to initiate these conversations [12,27,36,38].

Sociocultural influences further contributed to the under recognition of sexual health. Multiple studies described sexuality, aging, and death as intersecting taboos [35,49,50,51,55,56], reinforced by ageist assumptions, heteronormative perspectives, cultural beliefs, and misconceptions that individuals with advanced illness are no longer sexually active [26,36,38,57].

Interventions and Suggestions

Although there were no intervention studies as such, the recommendations were remarkably consistent across the literature. Most authors advocated incorporating routine sexual health assessment into PC consultations through brief screening questions or validated patient-reported outcome measures [21,26,27,45,53,54]. Structured communication models, particularly PLISSIT, were repeatedly recommended as practical frameworks to facilitate patient-centered discussions [26,36,57].

Professional education emerged as the most frequently proposed intervention. Nearly all studies emphasized the importance of improving clinicians' communication skills, increasing awareness of sexual health throughout advanced illness, broadening the concept of sexuality to include intimacy and emotional connection, and establishing referral pathways to psychosexual or sexology services whenever appropriate.

Implications for clinical practice

This review demonstrates that sexuality remains an integral dimension of quality of life for individuals receiving PC, despite the profound physical and psychosocial changes associated with advanced illness. Across all patient-focused studies [12,21,26,27,45,49,50,51], patients consistently reported changes in sexual desire, sexual activity, body image, and intimate relationships. However, rather than disappearing, sexuality frequently evolved into broader expressions of intimacy, including affection, emotional closeness, physical contact, and companionship. These findings support the contemporary understanding that sexuality transcends genital function or the sexual act and remains relevant throughout the course of serious illness [9,22].

The prevalence of qualitative studies describing affection, emotional intimacy, and relational closeness [49,51,54,56] suggests that traditional biomedical definitions of sexuality are insufficient to capture patient experiences in PC. As physical symptoms progress, patients often redefine intimacy, emphasizing non-sexual forms of connection that preserve emotional bonds and personal identity [26,35,54,56]. This shift reinforces previous conceptual frameworks proposing that sexuality should be approached as a multidimensional construct encompassing physical, psychological, relational, and existential dimensions, rather than being limited to reproductive or functional aspects [27,57]. Consequently, comprehensive PC should routinely acknowledge these broader dimensions when addressing quality of life [16,41,48,49,54].

Despite the importance patients attribute to sexuality [51,57], this review identified a considerable discrepancy between patient needs and routine clinical practice. In multiple studies, between three-quarters and over 90% of patients reported that healthcare professionals had never initiated conversations regarding sexuality or intimacy [12,21,26,27,53,54]. Interestingly, when such discussions did occur, patients generally perceived them as appropriate, helpful, and reassuring [12,27,51,53]. These findings indicate that the primary barrier may not be patient reluctance to discuss sexuality, but rather the lack of systematic assessment within routine PC consultations [35,36,38]. Similar conclusions have been reported in previous reviews, which maintain that sexuality-related concerns remain largely invisible because they are rarely incorporated into standard clinical assessments, despite their recognized contribution to general well-being [26,57].

This review also highlights that the limited assessment of sexuality reflects broader structural and organizational challenges within PC services. Healthcare professionals consistently described insufficient training, lack of confidence, personal discomfort, time constraints, and the prioritization of physical symptom management as the primary obstacles to addressing sexual health [26,35,36,38,52,57]. Furthermore, fragmented healthcare systems, high staff turnover, and limited continuity of care can hinder the development of therapeutic relationships characterized by trust and open communication [49,55]. Given that sexuality often requires sensitive and individualized conversations, the absence of longitudinal patient-professional relationships may further reduce opportunities for meaningful assessment. These findings suggest that improving communication requires not only individual training but also organizational strategies that foster continuity of care and integrate sexual health into routine assessment protocols [21,36,38,50,52,56].

Another significant finding concerns the lack of standardized approaches to assessing sexuality in PC; most quantitative studies utilized researcher-developed questionnaires, while qualitative studies predominantly employed semi-structured interviews, evidencing substantial methodological heterogeneity [4,50,51,52]. Among validated instruments, the EORTC QLQ-SH22 was the most frequently reported questionnaire, specifically within oncological cohorts [21,45]. Concurrently, communication models, such as PLISSIT, BETTER, 5As, ALARM, and Stepped Skills, were consistently recommended to facilitate patient-centered discussions [26,36,57,50]. However, none of these frameworks have been routinely implemented in palliative settings, and many professionals reported limited familiarity with their practical application [12,21,26,36,38,45]. Consequently, current evidence supports these models primarily as conceptual frameworks rather than as interventions with demonstrated clinical efficacy in PC.

The findings also underscore the influence of sociocultural factors on the inadequate recognition of sexuality. Various studies identified the persistence of misconceptions assuming that older adults or those living with advanced illness no longer experience sexual needs [35,38,49,50,51,53,57]. These beliefs are reinforced by ageism, cultural taboos regarding sexuality and death, and personal discomfort among professionals [36,52,54,55,56]. Such biases may inadvertently contribute to diminished quality of life by restricting opportunities for patients to express concerns related to sexuality, body image, or shifts in relationship dynamics [21,45,49,52,53]. Accordingly, addressing sexuality requires not only the enhancement of clinical competencies but also the critical examination of prevailing social and professional attitudes toward sexuality in PC.

The involvement of the partner also emerged as a significant aspect in the included studies, as the progression of illness often precipitates changes in relationship dynamics associated with physical limitations, treatment-related side effects, emotional distress, and role transitions, frequently resulting in feelings of rejection, loss of spontaneity, or uncertainty [21,26,27,45,49,54,58]. Nevertheless, several qualitative studies demonstrated that open communication regarding sexuality can facilitate mutual adaptation and strengthen emotional bonds despite the progression of physical deterioration [54,55]. These observations suggest that interventions focused exclusively on the patient may overlook critical relational dimensions of sexuality, supporting the inclusion of the partner, when appropriate, in comprehensive PC discussions [21,27,45].

Professional education emerged as the most consistently recommended strategy across the literature. Nearly all studies emphasized the importance of enhancing healthcare professionals’ communication skills, broadening their understanding of sexuality beyond sexual intercourse, incorporating routine assessment into clinical care, and establishing referral pathways to specialized psychosexual or sexology services when necessary [21,26,27,35,36,38,45,52,53]. These recommendations are consistent with the universal principles of PC, which advocate for holistic, person-centered attention. However, the total absence of intervention studies identified in this review highlights that current recommendations remain primarily based on expert opinion and observational evidence, rather than high-quality efficacy studies [26,36,57].

Limitations 

A major limitation of this review is the methodological heterogeneity of the included studies. Most were observational in design-cohorts, surveys, or qualitative analyses-with only two literature reviews and no interventional trials. This restricts the overall strength of evidence and limits causal interpretation, particularly where comparisons between the degree of sexual dysfunction and potential intervention strategies would have been valuable. Furthermore, many studies relied on relatively small sample sizes and employed diverse measurement scales, which complicates cross‑study comparisons and reduces generalizability. 

There is also a clear geographic and linguistic bias. Nearly all studies were conducted in high‑income countries, reducing applicability to low‑ and middle‑income settings. In addition, some publications appeared in non‑English languages, introducing potential cultural and linguistic bias. Also, since the authors are native Spanish speakers and the manuscript was written and revised in English, there is a potential linguistic bias. Population diversity was limited. Most studies focused on adults and older adults, with mean ages above 55 years. Only a few included younger patients or adolescents. Advanced cancer dominated the clinical focus, while other life‑limiting illnesses were less represented.

Finally, several practical and sociocultural barriers must be acknowledged. Outcomes relied heavily on self‑report, introducing bias. Clinical constraints such as lack of privacy, limited consultation time, and prioritization of physical symptoms restricted assessment. Sociocultural taboos around sexuality, aging, and death-reinforced by ageist and heteronormative assumptions-further contributed to underrecognition of sexual health.

Recommendations 

A central recommendation is the integration of routine sexual health assessment into PC consultations. This should be achieved through brief, sensitive screening questions or validated patient‑reported outcome measures such as the EORTC QLQ‑SH22. Normalizing sexuality as part of standard care would reduce the mismatch between patients’ expectations and clinical practice, where discussions remain rare. Importantly, HCP should actively raise this topic even when it is not the primary reason for the consultation, as the absence of such initiatives has been identified as a barrier to its integration.

Although there is not an algorithm standard, equally relevant is the adoption of structured communication models such as PLISSIT, BETTER, 5As, ALARM, and Stepped Skills, which provide practical frameworks for initiating and sustaining patient‑centered conversations. Their consistent recommendation across the literature underscores their potential value, although implementation remains limited. Approaches should also be tailored to age, gender, sexual orientation, and underlying pathology, as these factors significantly influence both sexual dysfunction and its management.

Another recurring recommendation is the need for professional education and training. Nearly all studies emphasized improving clinicians’ communication skills, increasing awareness of sexual health in advanced illness, and broadening the concept of sexuality to encompass intimacy and emotional connection. Training should also address personal discomfort and lack of confidence, which were frequently reported barriers. Incorporating sexual health education into undergraduate curricula, postgraduate training, and continuing professional development programs represents an essential step toward holistic, patient‑centered care.

Finally, the literature highlights the importance of referral pathways and sociocultural sensitivity. Clear systems for referral to psychosexual or sexology services would ensure that patients requiring specialized support receive appropriate care. At the same time, efforts must be directed toward challenging ageist assumptions, heteronormative perspectives, and cultural taboos that reinforce the invisibility of sexual health in PC. Promoting inclusivity and cultural sensitivity is crucial to ensure equitable recognition of sexual health needs across diverse patient populations.

It is recommended to conduct research with larger sample sizes, with both objective and subjective approaches, assessing the impact on quality of life through the implementation of protocols and guidelines for addressing sexual well-being by various HCPs. This assessment should correlate self-perception and sexual dysfunction grade, with stratification by groups based on underlying medical conditions and age groups.

## Conclusions

Sexual well-being is increasingly acknowledged in the literature as an important dimension of holistic PC, valued by patients and their partners. However, its integration into routine clinical practice remains limited. While in theory healthcare professionals recognize the relevance of sexuality and intimacy, in practice many avoid or minimize the topic due to discomfort, lack of training, time constraints, or sociocultural taboos. This gap between patient needs and professional practice highlights the persistent challenge of translating theoretical recognition into consistent clinical action.

On the other hand, there are obstacles within the healthcare setting, such as a lack of time during consultations, insufficient training and initiative among professionals to address these issues, or the lack of a private environment that fosters open communication about sexual health.

There is a need to develop protocols and rapid assessment tools aimed at the ongoing training of the palliative care team; their implementation will strengthen knowledge and provide staff with the confidence needed to address sexuality from both its biological and psycho-emotional dimensions. 
